# Let’s face it – 13 unusual causes of facial masses in children

**DOI:** 10.1007/s13244-015-0418-9

**Published:** 2015-07-20

**Authors:** Jacqueline du Toit, Nicole Wieselthaler

**Affiliations:** Department of Radiology, Red Cross Children’s Hospital, Klipfontein Road, Rondebosch, 7700 South Africa

**Keywords:** Facial neoplasm, Paediatric, Magnetic resonance imaging, Tumour-like, HIV

## Abstract

**Abstract:**

Facial swelling is commonly encountered in paediatric patients and is typically related to an underlying infection. The spectrum of possible causes, however, is wide, and includes traumatic, inflammatory, nutritional and neoplastic aetiologies. In this pictorial essay we present 13 examples of rare conditions selected from a total of 136 MRI examinations performed at our institution between April 2007 and May 2013. These include HIV-associated malignancies such as a case of plasmablastic lymphoma, parotid gland tumours including a parotid hamartoma, rare congenital lesions such as a thyroid fetiform teratoma, and infective lesions including tuberculosis of the mandible. In many cases, only minimal information could be gleaned from the literature, particularly with regard to imaging findings. An analysis of the spectrum of masses and specific clinical presentations allowed for the construction of a diagnostic flowchart which may serve to assist in unusual cases.

***Teaching Points*:**

• *Facial swelling is commonly encountered in paediatrics, with a wide spectrum of possible aetiologies.*

• *MRI is the favoured imaging modality for accurate assessment.*

• *Facial swelling is typically infectious in nature, but includes various benign and malignant causes.*

• *This pictorial essay presents 13 examples of rare conditions with corresponding imaging.*

## Introduction

Facial swelling is a common clinical scenario in the paediatric setting, associated with a diverse range of possible causes. The underlying aetiology ranges from congenital lesions to infective and inflammatory conditions, and various other benign or malignant masses [1].

Advances in imaging techniques have led to the ever-increasing use of computed tomography (CT) and magnetic resonance imaging (MRI) for evaluating the extent of disease and for treatment planning [1].

In the case of facial swelling associated with a systemic illness, poor response to antibiotic treatment, or a clinical suspicion of malignancy, cross-sectional imaging should be considered [2].

At our institution, MRI is favoured for the assessment of paediatric facial masses, with the advantages of superior soft tissue resolution and a lack of ionizing radiation.

There are several key clinical features that should be taken into account when interpreting this type of imaging, including the age of the child, the location of the mass, the duration and nature of onset, and any underlying condition associated with the development of neoplasms [3].

The most commonly encountered facial masses in the paediatric population are infective in nature, typically due to lymphadenitis, sinusitis, or a dental infection [1, 2].

Congenital masses tend to be non-progressive; examples include fronto-ethmoidal cephaloceles, nasal gliomas, and nasal dermoid and epidermoid cysts [1, 2].

Rapidly progressive masses include malignancies, with rhabdomyosarcoma and lymphoma being the most common, in addition to osteogenic sarcoma, Langerhans cell histiocytosis (LCH), Ewing’s sarcoma, and metastatic neuroblastoma [1, 4].

Although several reviews of the common causes of facial masses can be found in the literature, there is relatively little on the spectrum of more unusual causes such as salivary gland tumours and teratomas [4].

Our series includes 13 examples of rare, biopsy-proven conditions (Table 1), selected from a total of 136 MRIs of children presenting with facial masses between April 2007 and May 2013. These include two HIV-associated malignancies, three parotid gland tumours, two congenital facial masses, two lesions of the mandible, a paranasal sinus tumour, and three additional unusual facial malignancies. A literature review has been conducted, although in many instances the information available is scant.

**Table 1:** Thirteen paediatric patients presenting with unusual facial masses and investigated by MRI between April 2007 and May 2013

Patient	Age/Sex	Presentation	Management	Histological diagnosis	HIV status	Outcome
1	Newborn, F	Swelling over mandible at birth with associated stridor requiring tracheostomy	Surgical excision	Congenital thyroid fetiform teratoma	Unknown	Discharged
2	Newborn, F	Right facial mass at birth	Oncology	Congenital embryonal RMS	Unknown	Died aged 9 m
3	2y 7 m, F	Facial swelling left parotid region since birth	Surgical excision	Parotid hamartoma	Negative	Recurrence
	3y 5 m, F	Parotid swelling increasing in size since surgery	Bleomycin injection	Recurrence	Negative	Stable
4	7y 6 m, F	Right parotid swelling for 10 days	Abscess incision and drainage	Culture-confirmed TB mandible	Negative	Discharged upon completion of Rx
5	1y 6 m, F	Facial swelling left parotid region for 1 year	Surgical excision	Pilomatrixoma	Unknown	Discharged
6	10y 3 m, F	Swelling over central mandible for 1 year	Surgical excision	Giant cell granuloma	Unknown	Discharged
7	10y 8 m, F	Right facial swelling for 9 months	Patient elected return to local hospital for management	Maxillary schwannoma	Unknown	Uncertain
8	4 m, F	Left cheek swelling since 3 weeks of age	Surgical excision	Infantile fibrosarcoma	Negative (HIV exposed)	Oncology follow-up
9	4y 3 m, M	Right facial swelling for 1 month	Oncology	Parameningeal RMS	Unknown	Died aged 5y 4 m
10	1y 8 m, M	Left facial mass for 8 months	Oncology	Composite yolk sac and rhabdoid tumour	Unknown	Died aged 2y 1 m
11	11y 1 m, M	Generalised facial swelling for 1 month	Oncology, antiretrovirals	Plasmablastic lymphoma	Positive	Oncology follow-up
12	3y 8 m, M	Left jaw mass for 2 days	Oncology, antiretrovirals	Burkitt lymphoma	Positive	Oncology/Infectious Diseases follow-up
13	7y 1 m, F	Marasmic left parotid mass for 2 months	Oncology, antiretrovirals	Mucoepidermoid carcinoma	Positive	Oncology follow-up

A careful analysis of the various tumours and tumour-like lesions, as well as their clinical presentations, assisted in the design of a diagnostic flowchart (Table 2). Lesions present for 3 months or less were predominantly malignant tumours, but also included congenital and infectious causes. Those with a presence longer than 3 months were all benign, barring the malignant yolk sac tumour with rhabdoid elements, which was extremely advanced at presentation, and may therefore have been present for longer than the patient history suggested. The more ill-defined, destructive and invasive lesions tended to fall into the group of malignant tumours and infectious lesions, while the benign lesions tended to be well-circumscribed. The congenital lesions comprised both benign and malignant aetiologies and thus exhibited variable imaging characteristics.

**Table 2:**
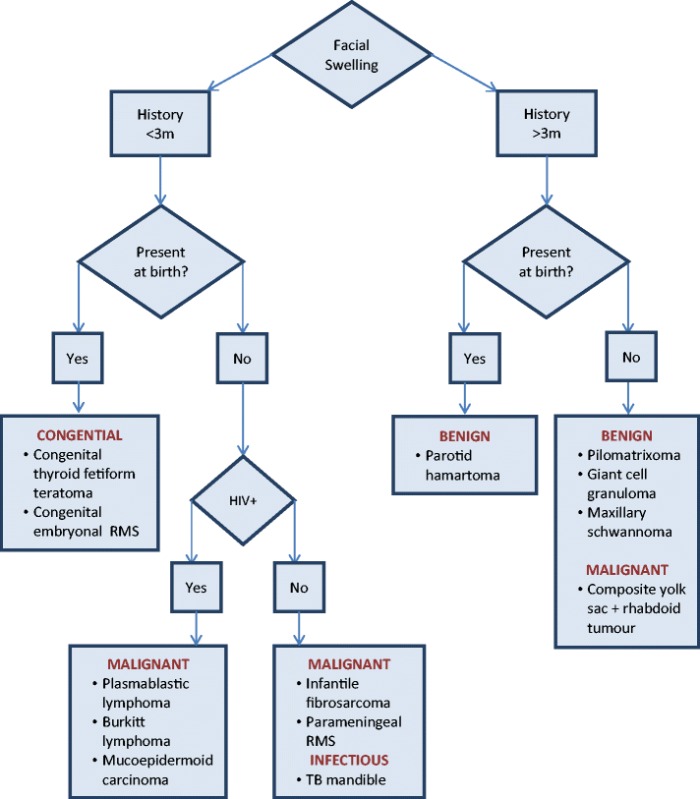
Diagnostic flowchart for paediatric patients presenting with unusual facial massesᅟ

## Congenital lesions

### Fetiform teratoma

Fetiform teratoma is a rare form of highly developed cystic teratoma which resembles a malformed fetus. Only very few cases have been reported in the literature, most presenting as ovarian masses in women of reproductive age [5, 6]. The reported age range, however, does span the neonatal period to age 65 [5]. No previously described case of a facial fetiform teratoma could be identified in the literature.

Although fetiform teratomas develop a high degree of differentiation and organisation, they do not typically contain complex, well-developed organ systems [6].

The tumour must be distinguished from fetus-in-fetu, which results from the inclusion of a monochorionic diamniotic twin within its host twin [7]. Diagnosis of fetus-in-fetu requires the presence of a highly developed and segmented axial skeleton, as well as organogenesis [8].

The degree of development and organisation can vary, however, blurring the distinction between the two entities, and it has been suggested that a continuum may exist [8].

MRI signal characteristics have been described as heterogeneous, with central areas of T2 hypointensity consistent with developed bony components, and adjacent hyperintensity representing fat and soft tissue components [5].

In our case, the child presented with a swelling over the right mandible at birth and associated stridor requiring tracheostomy.

**Fig. 1:**
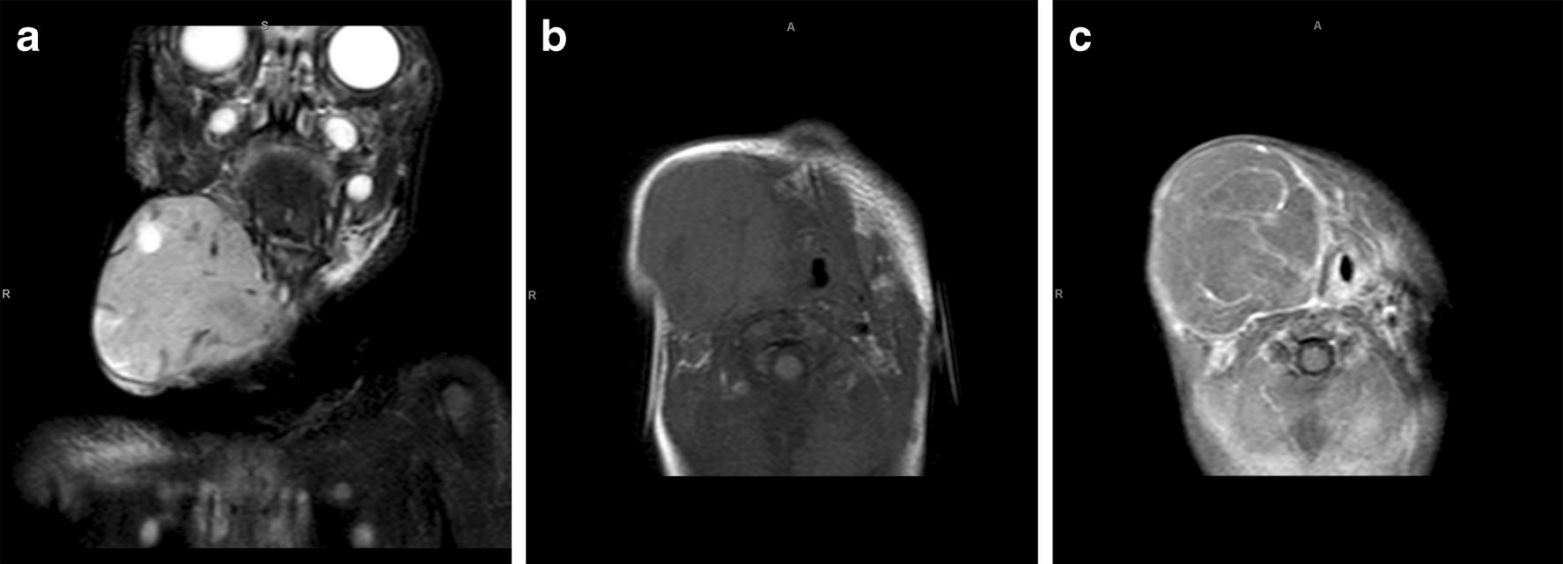
Fetiform teratoma. Coronal T2-W (**a**) and axial T1 W (**b**) MRI performed at 1 month of age shows a well-defined solid mass medial to the ramus of the mandible on the right. There are small hypointense foci on the T2-W image (**a**), and the mass enhances poorly post-contrast (**c**)

MRI performed at 1 month of age showed a well-defined solid mass medial to the ramus of the mandible on the right. There were small hypointense foci on T2-weighted imaging, and the mass enhanced poorly post-contrast (Fig. 1).

### Congenital rhabdomyosarcoma

Rhabdomyosarcoma (RMS) is an aggressive malignant soft tissue neoplasm of skeletal muscle origin which accounts for up to 8 % of all malignancies in children under 15 years of age. Neonatal presentation of this tumour, however, is rare [9].

A large cohort analysed by the IRS group showed that only 0.4 % presented before the age of 1 month [9].

The diagnosis of congenital RMS suggests the possible intrauterine development of this tumour, and the head, neck, and trunk are the most commonly affected sites [9].

On MRI, the tumours are typically hypointense to skeletal muscle on T1-weighted (T1-W) and hyperintense on T2-weighted (T2-W) imaging, enhance heterogeneously post-contrast, and demonstrate prominent vascularity [10].

**Fig. 2:**
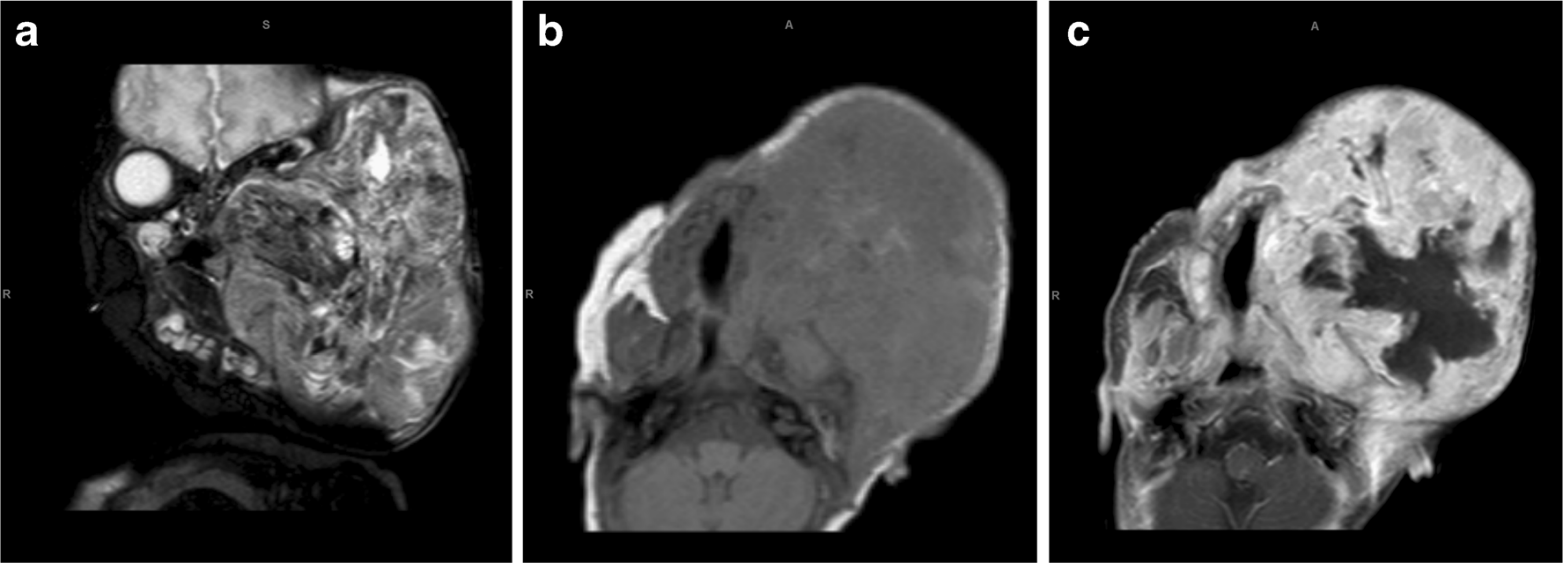
Congenital rhabdomyosarcoma. Coronal T2-W (**a**) and axial T1-W (**b**) MRI images show a 10 × 10.5 cm heterogeneous mass centred within the left parapharyngeal soft tissues. The tumour extends from the middle cranial fossa to the level of C4 inferiorly, with associated destruction of the left ramus of the mandible and the left lateral orbital wall. On the post-contrast T1-W image (**c**), there is avid enhancement with a large area of necrosis centrally

Our MRI, performed when the baby was 5 days old, showed a 10 × 10.5 cm heterogeneous mass centred within the left parapharyngeal soft tissues. The tumour extended from the middle cranial fossa to the level of C4 inferiorly. There was associated destruction of the ramus of the left mandible and left lateral orbital wall. On the post-contrast images, there was avid enhancement with a large area of necrosis centrally (Fig. 2).

### Parotid hamartoma

Hamartomas of the parotid gland are exceedingly rare, with only four cases having been described in the English language literature [11].

These benign tumour-like lesions arise during the development phase of an organ or tissue, and consist of a disorganized growth of differentiated mature tissues indigenous to that particular anatomic location [11].

No description of imaging findings of this rare lesion could be located in the literature.

**Fig. 3:**
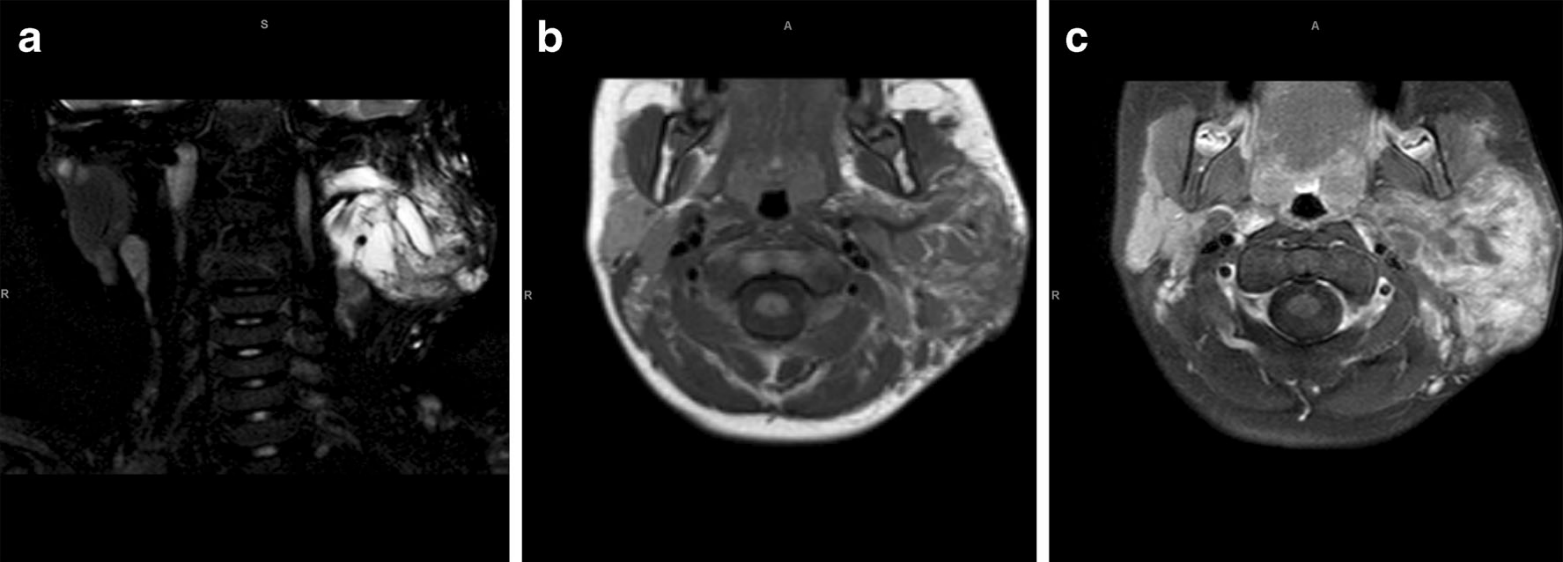
Parotid hamartoma. Coronal T2-W MRI (**a**) shows the left parotid gland to be expanded and almost entirely replaced by a heterogeneous, mixed solid and cystic lesion which is mixed intensity on the axial T1-W view (**b**) and enhances avidly post-contrast (**c**)

In our example, the left parotid gland was expanded and almost entirely replaced by an avidly enhancing, heterogeneous mixed solid and cystic lesion (Fig. 3).

## Infectious lesions

### TB mandible

Tuberculosis (TB) is a chronic granulomatous disease caused by Mycobacterium tuberculosis. Although the chest is most frequently affected, any organ system may be involved, particularly in immunocompromised individuals [12].

Musculoskeletal tuberculosis, however, accounts for only 1–3 % of cases [12], with TB of the mandible being rare and constituting only 2 % of these [13].

Radiologically, tuberculous osteomyelitis typically appears as a unilocular destructive bone lesion with an associated periosteal reaction [14].

**Fig. 4:**
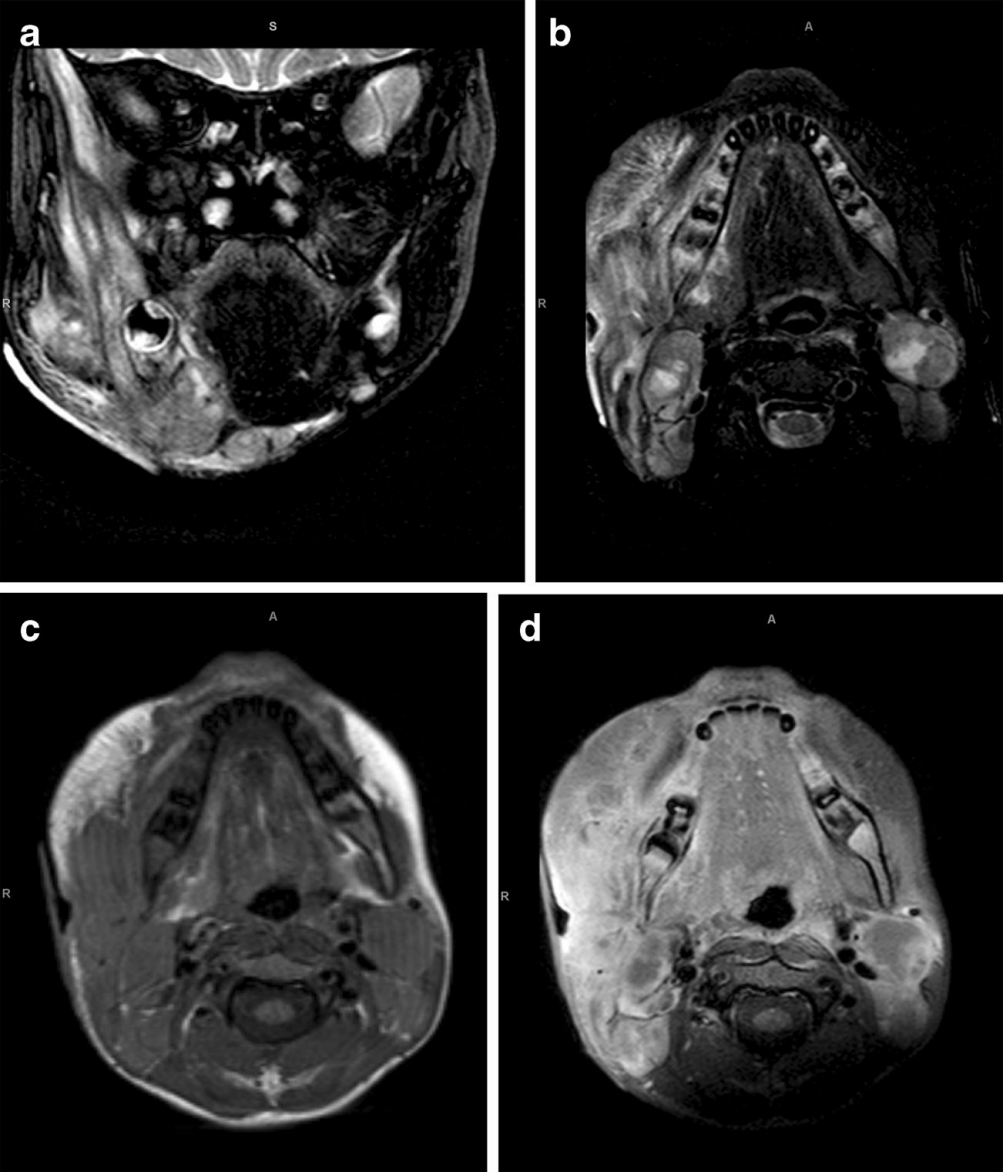
TB mandible. Coronal (**a**) and axial (**b**) T2-W MRI images show a destructive right mandibular lesion involving the ramus and coracoid process, with a periosteal reaction and associated soft tissue mass. The pre- (**c**) and post-contrast (**d**) T1-W images demonstrate heterogeneous enhancement of the soft tissue component. Characteristic of TB are large bilateral carotid space nodes which are predominantly T2 hypointense (**b**), with rim enhancement and central necrosis (**d**)

Our MRI showed a destructive bone lesion of the right mandible involving the ramus and coracoid process, with a periosteal reaction and associated heterogeneously enhancing soft tissue mass. Characteristic of TB were large bilateral carotid space nodes which were predominantly T2 hypointense, with rim enhancement and central necrosis (Fig. 4).

## Benign tumours

### Pilomatricoma

Pilomatricoma is a benign skin neoplasm derived from hair follicle matrix cells [15] and is commonly misdiagnosed, with imaging features that are not well understood [16].

Clinically, pilomatricomas often present as a solitary, superficial, rock-hard mass—a pathognomonic feature—with the overlying skin occasionally demonstrating a bluish discolouration [15, 17]. Although pain and secondary infection may occur, the majority of lesions tend to be asymptomatic, as was the case in this example.

Pilomatricomas occur most commonly in the first and second decades of life, predominantly in female patients—as was our patient [15].

Despite a lack of agreement regarding imaging features, pilomatricoma has been described as having a uniform intermediate signal on T1, heterogeneous signal on T2, and internal reticulations on contrast-enhanced T1-W images corresponding to oedematous stroma [16]. Peripheral enhancement has also been described [16].

**Fig. 5:**
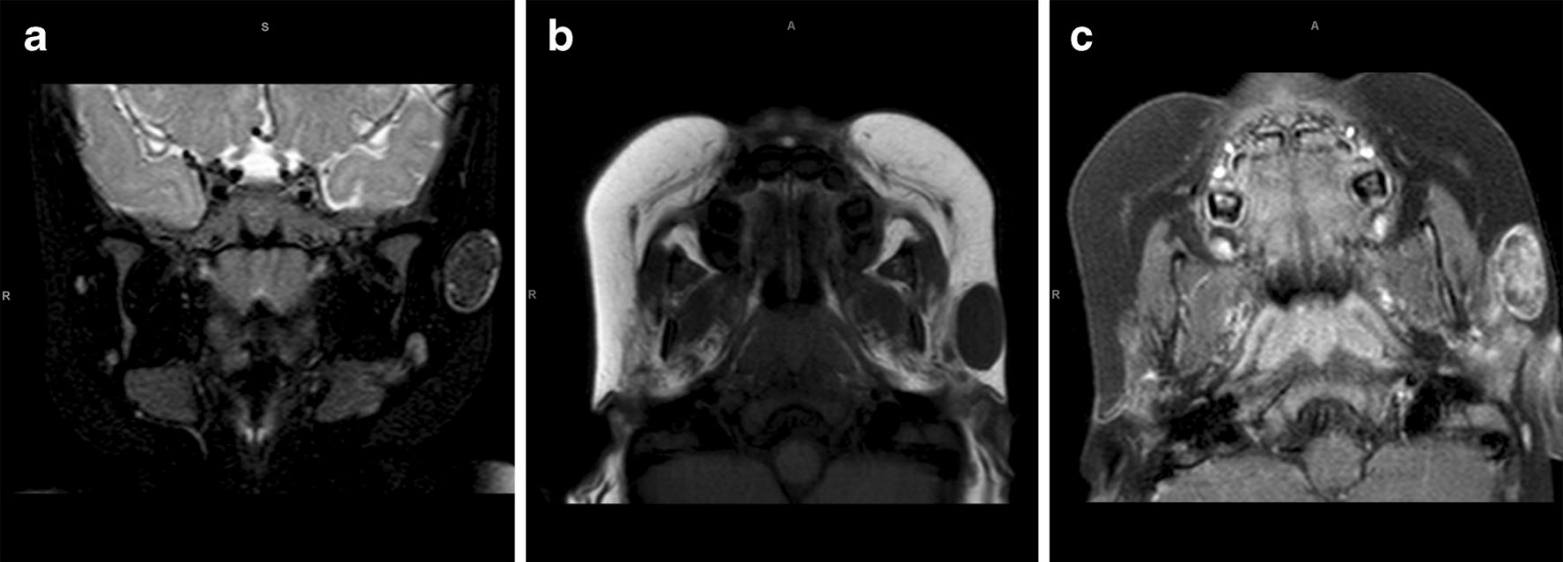
Pilomatricoma. Coronal T2-W (**a**) and axial T1-W (**b**) MRI shows a hypointense 23 × 12 mm subcutaneous nodule on both views, with a well-defined T2 hyperintense rim (**a**). On the T1-W post-contrast view (**c**), the rim enhances avidly and the central component heterogeneously

Our MRI showed a 23 × 12 mm T1 and T2 hypointense subcutaneous nodule, with a well-defined T2 hyperintense rim. On the post-contrast images, there was both rim enhancement and diffuse reticular enhancement centrally (Fig. 5).

### Central giant cell granuloma

Central giant cell granulomas (CGCGs) have been described as uncommon benign bone lesions, typically affecting the mandible and maxilla [18]. There is considerable controversy regarding the exact aetiology, which may be that of a reactive lesion following an episode of intraosseous haemorrhage or inflammation, or alternatively, a true neoplasm related to giant cell tumours [18, 19].

There is a wide age range affected, but the majority occur in patients younger than 30 years, and there is a 2:1 female predilection [19]. Our female patient presented at age 10 years.

The lesions exhibit a wide spectrum of clinical behaviour, ranging from a painless, slowly-progressive swelling, to a larger, locally-destructive ‘aggressive’ lesion with a higher rate of recurrence following excision and curettage [18].

Similarly, there is considerable variation in the imaging findings. The spectrum ranges from a small unilocular lesion, to a much larger lesion with ill-defined margins, multiple loculations, and associated tooth displacement or root resorption [18].

**Fig. 6:**
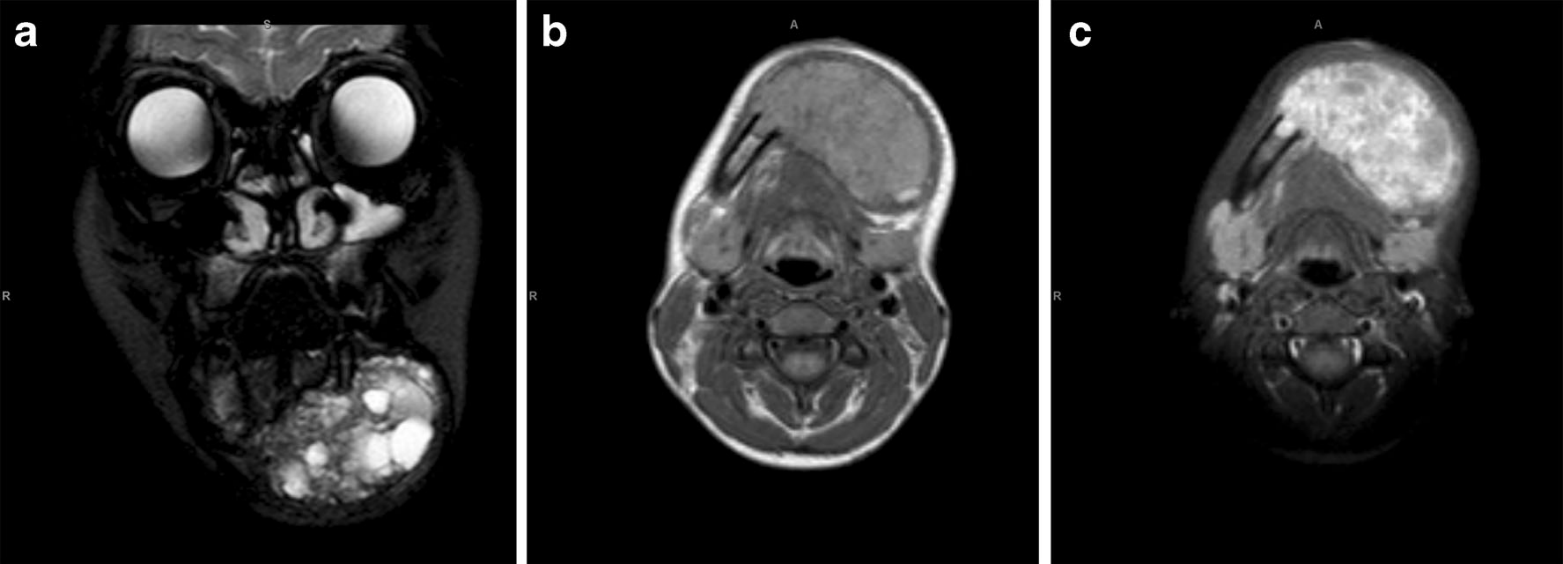
Central giant cell granuloma. Coronal T2-W (**a**) and axial T1-W (**b**) MRI show a well-corticated, multi-cystic, expansile mandibular lesion, with solid components which enhance avidly post-contrast (**c**)

Our imaging revealed a well-corticated, multi-cystic, expansile mandibular lesion, with solid components which enhanced avidly post-contrast (Fig. 6).

### Maxillary schwannoma

A schwannoma (neurilemmoma) is a benign, slow-growing, encapsulated perineural tumour arising from nerve sheath Schwann cells [20].

Schwannomas occurring in the head and neck region are not unusual, but those originating in the maxillary sinuses are rare, with only seven cases to date having been reported in the literature [1, 21].

No case occurring in a child could be found in the literature, with the youngest reported case in a patient 17 years of age [1].

Clinically, the lesions are typically asymptomatic, but may present with pain, an enlarging mass, or proptosis [1], as was the case in this example.

No description of typical MRI features could be found.

**Fig. 7:**
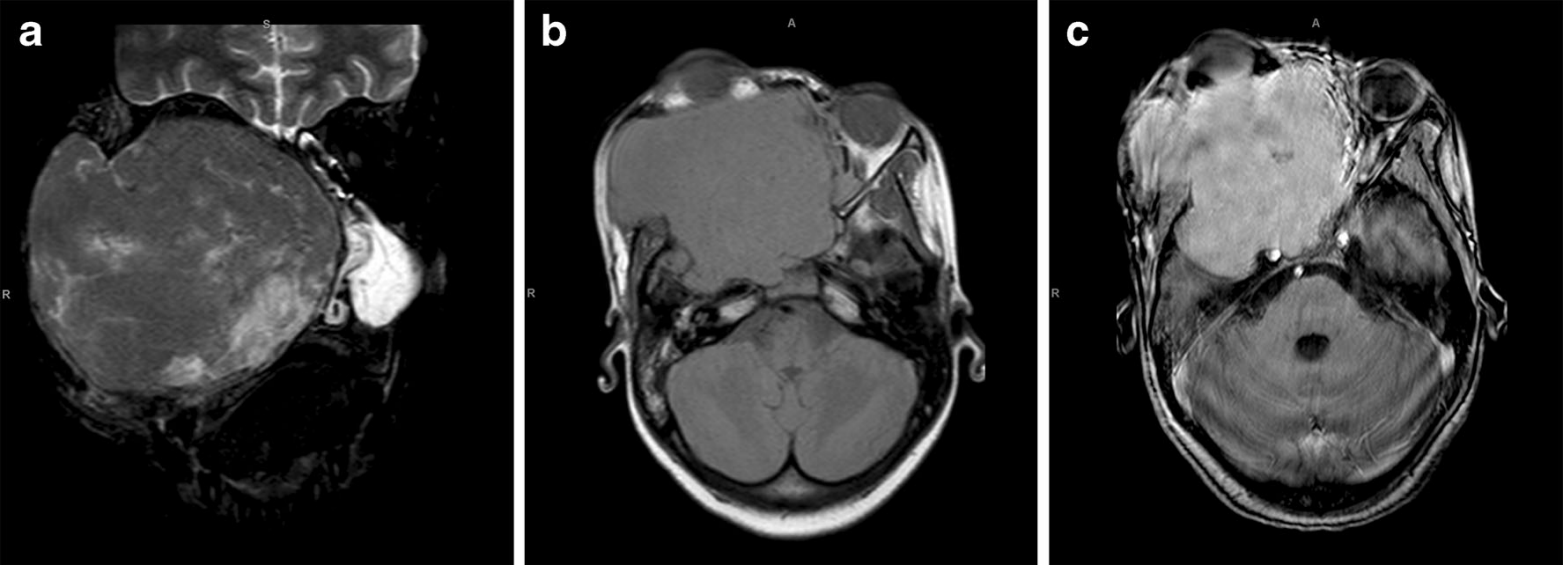
Maxillary schwannoma. Coronal T2-W (**a**) MRI shows a large tumour centred in the right maxillary sinus, with associated proptosis. The heterogeneous signal hypointensity implies high cellularity. The mass is iso-intense to grey matter on the T1-W view (**b**) and enhances post-contrast (**c**)

Our case demonstrated a lesion with heterogeneous hypointense signal on T2, implying high cellularity. The mass was iso-intense to grey matter on T1 and enhanced post-contrast (Fig. 7).

## Malignant tumours

### Infantile fibrosarcoma

Infantile fibrosarcoma is a rare soft tissue neoplasm diagnosed at birth or soon afterwards.

These tumours typically affect the distal extremities, with only 16 % occurring in the head and neck region [22], and are often clinically misdiagnosed as hemangiomas [23].

The lesions may become disproportionately large relative to the size of the child, and tumours as large as 30 cm have been reported [22].

On MRI there is typically a heterogeneously enhancing soft tissue mass containing focal areas of hypo-enhancement due to haemorrhage or necrosis [22].

In our example, MRI showed a left subcutaneous masticator space lesion which was avidly enhancing and contained foci of low signal consistent with central necrosis.

**Fig. 8:**
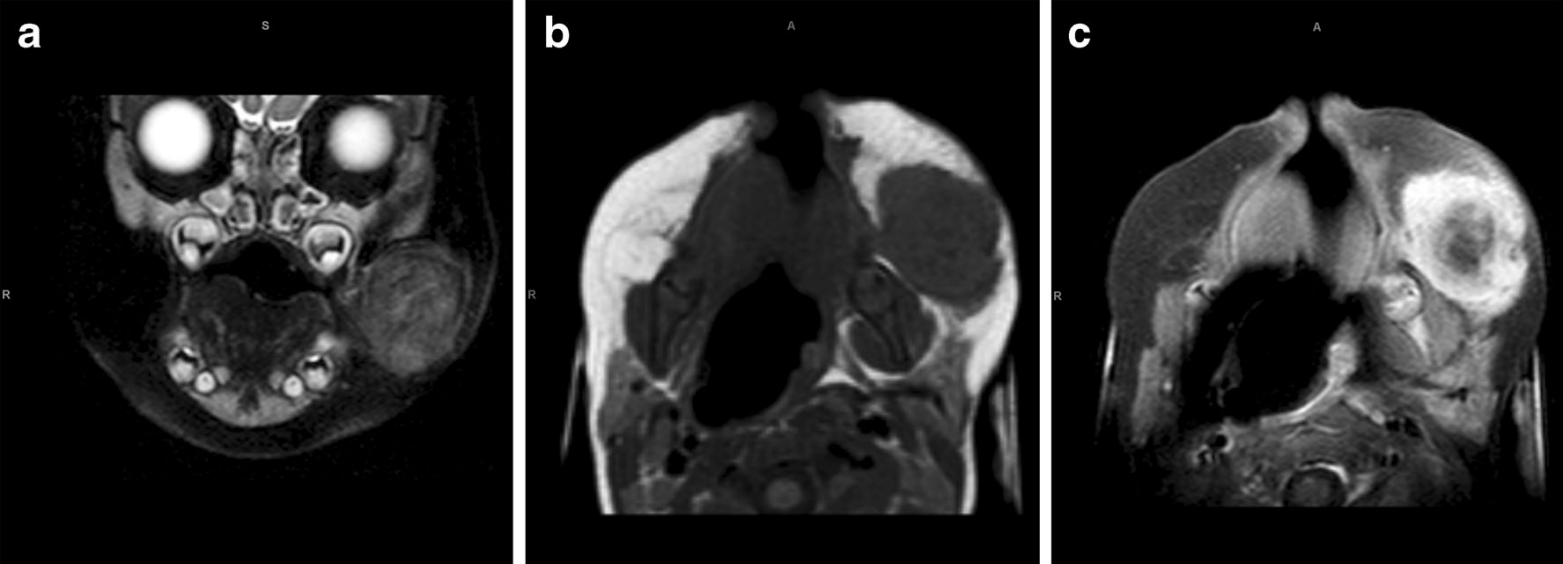
Infantile fibrosarcoma. Coronal T2-W (**a**) and axial T1-W (**b**) MRI images show a subcutaneous left masticator space lesion. The mass is avidly enhancing post-contrast (**c**) and contains foci of low signal consistent with central necrosis

There were no significant intralesional flow voids to suggest a vascular malformation (Fig. 8).

### Parameningeal rhabdomyosarcoma

Rhabdomyosarcomas (RMSs)—which originate from primitive mesenchymal tissue—constitute between 3 and 5 % of childhood malignancies [24]. The region most commonly affected is the head and neck, accounting for approximately 35 % of cases. This is followed by the genitourinary tract and the extremities [9].

Based on their location in the head and neck region, they may be classified as 1) orbital, 2) parameningeal, or 3) non orbital-non parameningeal [9]. The parameningeal sites include the pterygopalatine and infratemporal fossae, paranasal sinuses, middle ear, and mastoid. These tumours have a tendency toward local and intracranial extension [24].

The clinical and biological behaviour of RMSs varies widely with their appearance, ranging from a small cutaneous facial nodule to an extensive rapidly progressive facial swelling [9], as was the case with our patient.

Our patient, a 4-year-old child, presented with an apparent history of progressive facial swelling over the previous month. On examination, there was a massive right-sided exophytic tumour with a destroyed right eye.

**Fig. 9:**
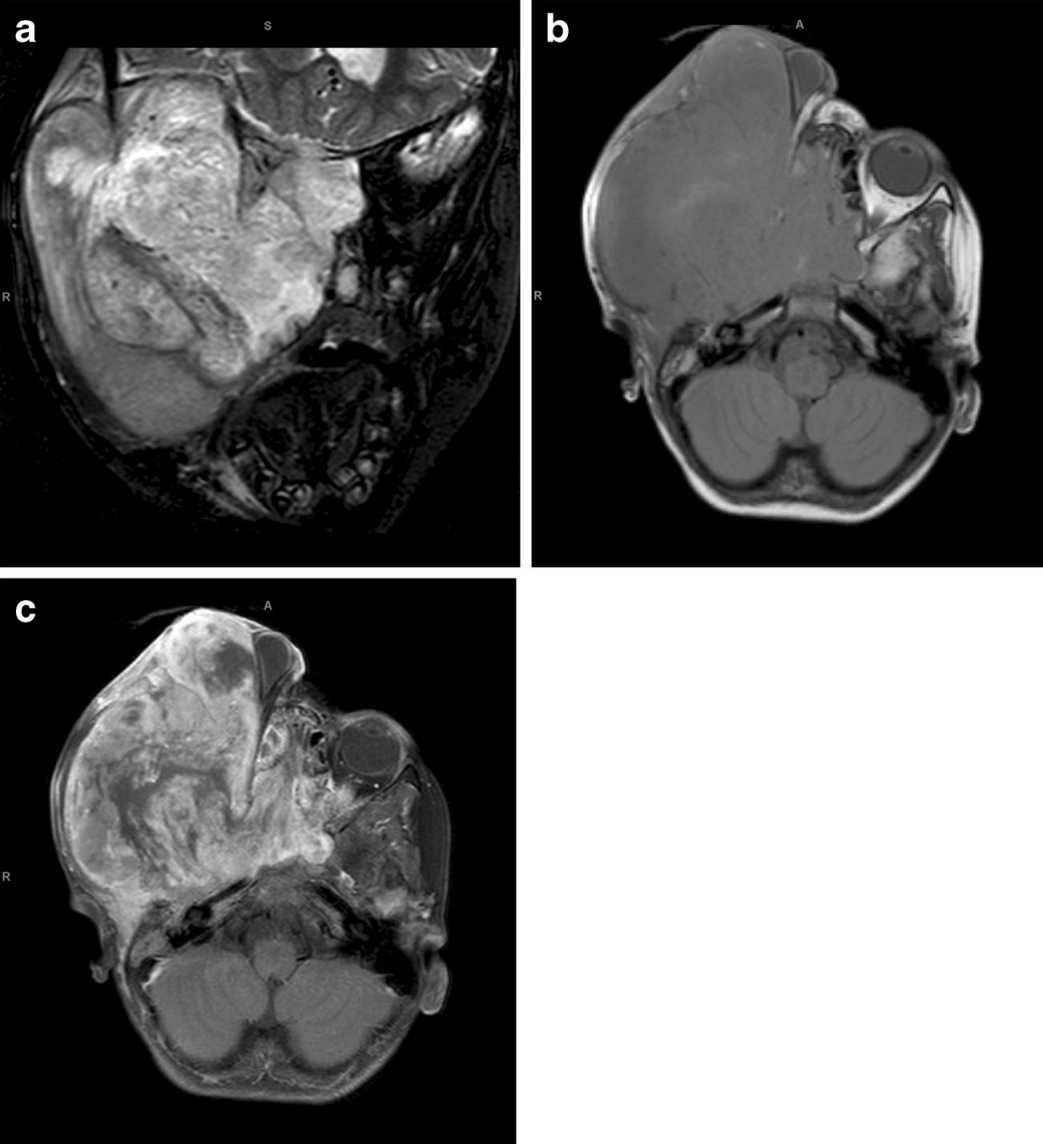
Parameningeal rhabdomyosarcoma. Coronal T2-W (**a**) and axial T1-W (**b**) images show a 12 × 10 cm lobulated, heterogeneously enhancing (**c**) right facial mass extending from the base of skull to the inferior margin of the mandible, with invasion of the infratemporal fossa and destruction of the right globe

MRI showed a 12 × 10 cm aggressive lobulated heterogeneous right facial mass extending from the base of the skull to the inferior margin of the mandible, with invasion of the infratemporal fossa and destruction of the right globe (Fig. 9).

### Yolk sac tumour with rhabdoid elements

Yolk sac tumours (YSTs) are malignant neoplasms of germ cell origin, usually occurring in the ovary or testes. Extragonadal YSTs are uncommon and most often seen in the sacrococcygeal, mediastinal, intracranial, and retroperitoneal regions. Extracranial head and neck YSTs are exceedingly rare [25].

YSTs primarily affect neonates and infants, and often occur in conjunction with other germ cell tumours, most commonly teratomas [26].

In the head and neck, extracranial YSTs may involve the orbit, maxillary sinus, retroauricular region, oral cavity, nasopharynx, submandibular gland, and parotid gland [26].

YSTs tend to be very aggressive and have early metastatic potential, usually involving the lungs, lymph nodes, liver, and bones [26].

MRI features have been described as a well-defined hyperintense tumour [25].

**Fig. 10:**
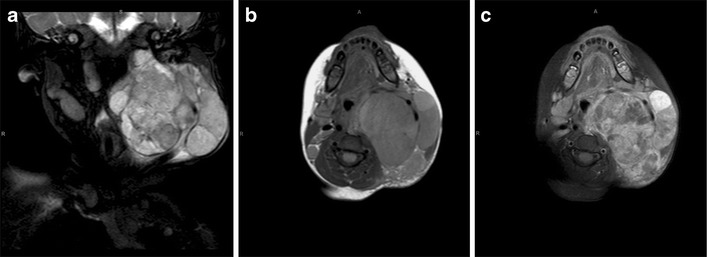
Yolk sac tumour with rhabdoid elements. Coronal T2-W (**a**) and axial T1-W (**b**) MRI show a large well-circumscribed left cervical mass centered in the parapharyngeal space, extending from the skull base to the level of the thoracic inlet, with associated right cervical adenopathy. The mass is predominantly hyperintense on T2-W imaging (**a**) and demonstrates heterogenous post-contrast enhancement (**c**)

In our case, MRI revealed a large well-circumscribed left cervical mass centred in the parapharyngeal space, extending from the skull base to the level of the thoracic inlet, with associated right cervical adenopathy. The mass was predominantly hyperintense on T2 and demonstrated heterogeneous post-contrast enhancement (Fig. 10).

## Malignant tumours in HIV

### Plasmablastic lymphoma

Plasmablastic lymphoma (PBL) has been classified by the WHO as a new clinical entity, and characterised as an aggressive, invariably fatal, subtype of non-Hodgkin’s lymphoma, typically occurring in HIV-infected patients [27, 28].

Epstein–Barr virus and Kaposi sarcoma-associated human herpesvirus-8 are also thought to play a role in the pathogenesis of PBL [27].

PBL is a rare phenomenon in children, with only 10 cases reported in the literature [29–31]. Our case is that of an 11-year-old HIV-infected boy.

Although originally described as a disease arising in the oral cavity of immune-deficient patients, subsequent cases involving extraoral sites such as the maxillary sinus, nasopharynx, lung, skin, anus, and spermatic cord have been reported [27].

A description of the typical imaging features could not be found in the literature. Our MRI showed multiple bilateral lobulated facial masses which were T2 hypointense with multi-septated rim enhancement.

**Fig. 11:**
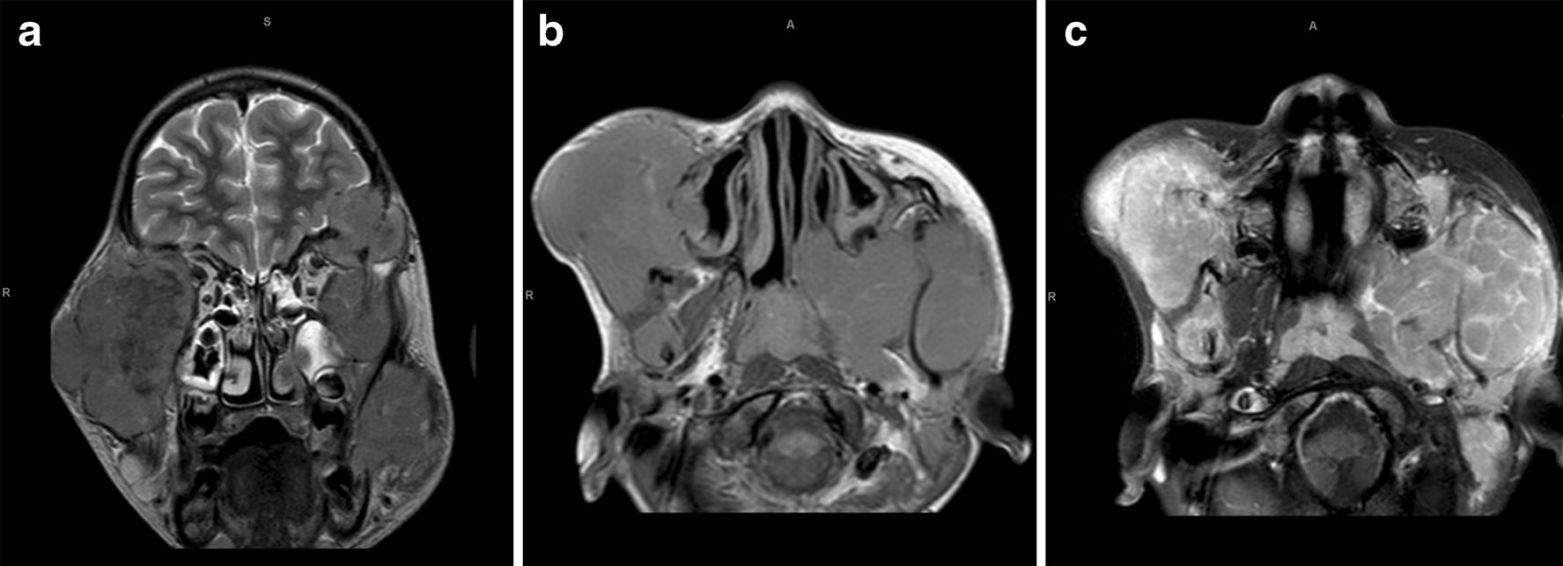
Plasmablastic lymphoma. Coronal T2-W MRI (**a**) shows multifocal bilateral lobulated facial masses which are predominantly hypointense. On the axial T1-W pre- (**b**) and post-contrast (**c**) images there is multi-septated rim enhancement

Mortality at 1 year is said to be approximately 60 %, although the combination of highly active antiretroviral therapy (HAART) and chemotherapy may significantly improve the prognosis [27]. At the time of publication, our patient was alive and undergoing oncology follow-up (Fig. 11).

### Burkitt lymphoma

Burkitt lymphoma—an undifferentiated non-Hodgkin lymphoma—is an AIDS-defining illness and a commonly encountered AIDS-related malignancy [32].

Epstein–Barr virus has been associated with approximately half of AIDS-related Burkitt lymphoma cases [32].

In a study comparing HIV-positive children admitted with malignancy to HIV-negative children with malignancy, Burkitt lymphoma was found to occur 7.2 times as frequently in the HIV-positive group [33].

Although HIV-associated Burkitt lymphoma typically involves the abdominal organs and bone marrow, our patient presented with a left-sided mandibular mass.

**Fig. 12:**
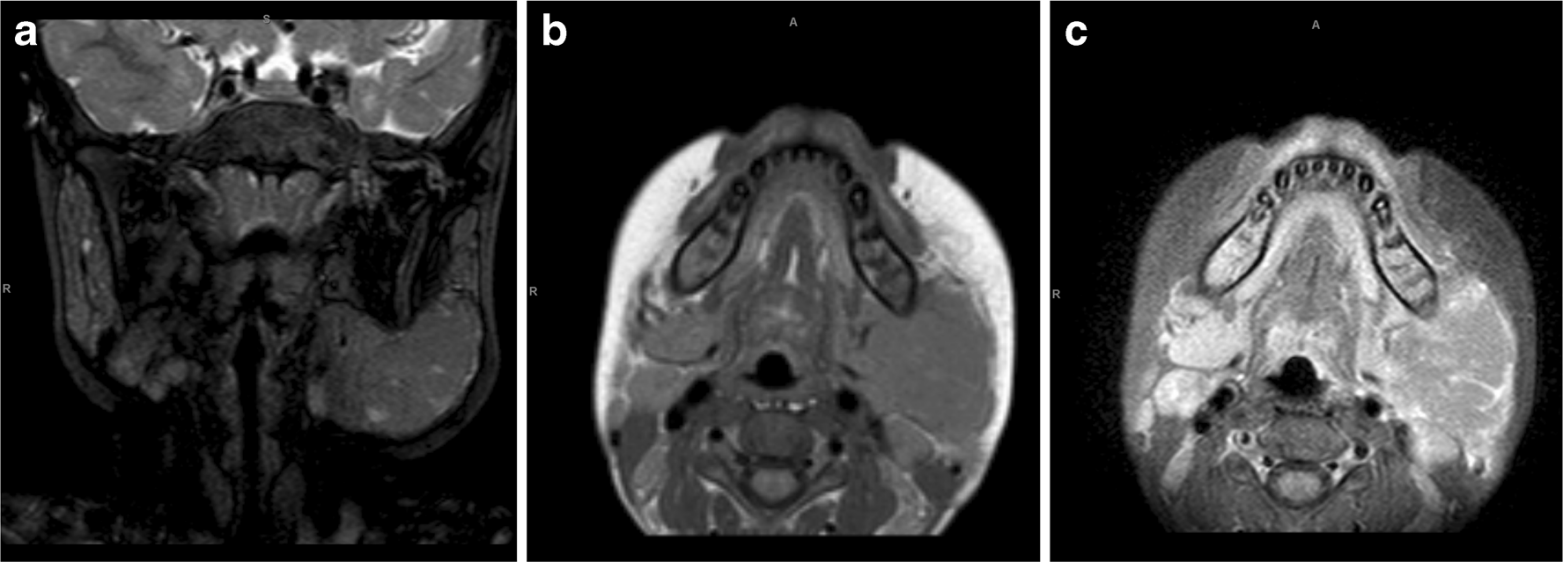
Burkitt lymphoma. Coronal T2-W (**a**) and T1-W pre-contrast (**b**) MRI show a subcutaneous mass posterior to the left mandibular angle which is iso-intense on both sequences. On the axial T1-W post-contrast scan (**c**), there is heterogeneous enhancement and associated posterior triangle lymphadenopathy

MRI revealed a T1 and T2 iso-intense subcutaneous mass posterior to the left mandibular angle, with heterogeneous enhancement post-contrast and associated posterior triangle lymphadenopathy (Fig. 12).

### Mucoepidermoid carcinoma

Malignant parotid gland tumours are very rarely observed in children, particularly those under 10 years of age [34, 35].

A review of 122 paediatric patients with salivary gland tumours found only 17 to be malignant. All 17 occurred between the ages of 11 and 18 [35], while our patient presented at just 7 years of age.

According to Belghiti et al., fewer than 19 cases of malignant parotid gland tumours in children have been published, with mucoepidermoid carcinoma accounting for approximately one third of these [34].

Although a clear link between mucoepidermoid carcinoma and HIV has yet to be established, Serraino et al. described an increased age-standardised incidence of salivary gland cancer in HIV-positive adult men compared with HIV-negative men [36].

Imaging appearance is linked to histological grade, ranging from a well-circumscribed heterogeneous parotid space mass to a more invasive, ill-defined tumour with associated lymphadenopathy [37].

**Fig. 13:**
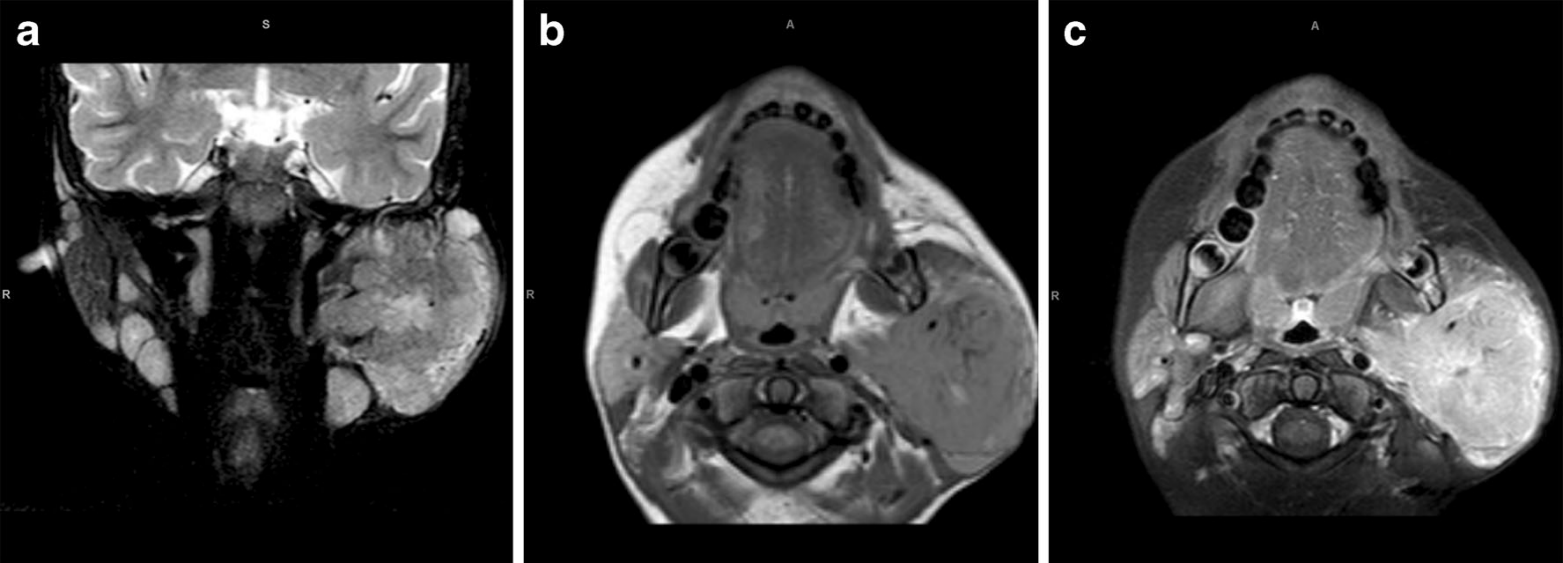
Mucoepidermoid carcinoma. Coronal T2-W MRI (**a**) shows a hyperintense, well-defined left intra-parotid mass which is iso- to hyperintense on the axial T1 W view (**b**), and enhances avidly post-contrast (**c**). There are intratumoral cysts and necrosis, as well as associated bilateral cervical and posterior triangle lymphadenopathy

In our example, the left intra-parotid mass was well-defined, hyperintense to muscle on T2, iso- to hyperintense on T1, and enhanced avidly post-contrast. There were intratumoral cysts and necrosis as well as bilateral cervical and posterior triangle lymphadenopathy (Fig. 13).

## Conclusions

Although infective causes account for the majority of cases of paediatric facial masses or mass-like lesions, there is a much wider spectrum of more unusual aetiologies, which have been highlighted in this pictorial essay, and to which radiologists should be alerted in order that appropriate treatment is not delayed.
